# Scrolling through adolescence: unveiling the relationship of the use of social networks and its addictive behavior with psychosocial health

**DOI:** 10.1186/s13034-024-00805-0

**Published:** 2024-08-31

**Authors:** Caroline Brand, Camila Felin Fochesatto, Anelise Reis Gaya, Felipe Barreto Schuch, José Francisco López-Gil

**Affiliations:** 1 Physical Education School, IRyS Group, Pontificia Universidad Católica de Valparaíso, Avenida El Bosque 1290, Sausalito, Viña del Mar, Valparaíso, Chile; 2 School of Physical Education, Physiotherapy and Dance, Federal University of Rio Grande do Sul, Felizardo Street, 750, Porto Alegre, RS Brazil; 3 Department of Sports Methods and Techniques, Federal University of Santa Maria, Santa Maria, Brazil; 4 Institute of Psychiatry, Federal University of Rio de Janeiro, Rio de Janeiro, Brazil; 5 Faculty of Health Sciences, Universidad Autónoma de Chile, Providência, Chile; 6 One Health Research Group, Universidad de Las Américas, Quito, Ecuador

**Keywords:** Psychosocial health, Mental health, Screen time, Youth, Teenagers, Mobile phones

## Abstract

**Background:**

Understanding the relationship of social network use and addictive behaviors with adolescent psychosocial health is crucial in today’s digital age.

**Aim:**

To verify the associations between social network use, messaging applications, and the addictive behaviors to social network with psychosocial health in Spanish adolescents.

**Methods:**

A cross-sectional study was developed with 632 adolescents, aged 12 to 17 years from the Region of Murcia, Spain. The assessment of social network use (Facebook, Twitter, Instagram, Snapchat, and TikTok) involved evaluating the frequency of use of each social network individually using a single-item scale with five response options. WhatsApp use (i.e., a messaging application) was evaluated in the same manner. The Short Social Networks Addiction Scale-6 Symptoms was employed to assess potential addictive behaviors to social network use. The psychosocial health was assessed using the Strengths and Difficulties Questionnaire. Generalized linear regression models were conducted, and predictive probabilities of having psychosocial health problems were calculated.

**Results:**

The predicted probability of presenting psychosocial health problems in the medium users and high users of social networks was 19.3% (95% confidence interval [CI] 13.0 to 27.7), and 16.2% (95% CI 10.2 to 24.6) higher compared to low users, respectively. High usage of Instagram, TikTok, Snapchat, and Facebook was associated with increased probabilities of psychosocial health problems, with Facebook showing the highest probabilities, at 31.3% (95% CI 14.8 to 54.2) for medium users and 51.9% (95% CI 26.5 to 76.3) for high users. Additionally, adolescents with addictive behaviors to social network use had from 19.0 to 25.2% probabilities of experiencing psychosocial health problems. Finally, the highest probabilities of having psychosocial health problems were identified in adolescents with high addictive behaviors when using social networks (28.9%; 95% CI 19.3 to 40.8%) and the lowest in those with low addictive behaviors (6.8%; 95% CI 3.3 to 13.6%).

**Conclusion:**

Adolescents who use social networks more frequently and exhibit more addictive behaviors related to their use are more likely to experience psychosocial health problems compared to those who do not. Facebook showed the strongest association, followed by Snapchat, Instagram, and TikTok. Our data also revealed that adolescents exhibit various signs of addictive behaviors to social network use.

**Supplementary Information:**

The online version contains supplementary material available at 10.1186/s13034-024-00805-0.

## Introduction

In recent years, there has been a notable increase in media device usage, with estimates indicating that between 88 and 95% of American adolescents now own smartphones [1], and almost 90% are online at least several times during the day [2]. Consequently, the utilization of social networks and messaging applications, such as Instagram, TikTok, and WhatsApp, among adolescents, has become a prominent aspect of contemporary youth culture [3]. This trend has significantly influenced their social interactions and communication patterns. Social networks offer adolescents opportunities for self-expression, social connection, and information sharing. Similarly, messaging applications like WhatsApp facilitate instant communication and group interactions among peers [4]. However, alongside the benefits, concerns have emerged regarding the potential impact of excessive social network use and WhatsApp usage on adolescent psychosocial health [5–7].

The use of social media is a contentious issue, given the presence of mixed evidence regarding its impacts. Mountain cross-sectional evidence has indicated that excessive social network use is associated with higher symptoms of anxiety, depression, hyperactivity, and conduct problems among adolescents [7–12]. For instance, a study involving 6596 US adolescents revealed that spending more than 3 h per day on social networks is linked to a higher probability of mental health issues [8]. Also, a longitudinal study revealed an association between problematic social network use and later anxiety symptoms in adolescents aged 13 to 14 years [6]. However, results are not entirely consistent, demonstrating that more time spent on social media was not significantly related to poorer mental health 2 years later in adolescents [13]. Also, evidence suggests that the use of Facebook may have more positive than negative effects on mental health [14].

In this context, it is important to highlight that excessive social network use can lead to behavioral addiction [15], impacting various aspects of an individual’s life, including academic performance and overall well-being [16, 17]. Moreover, the addictive nature of social network use among adolescents raises concerns about the long-term implications on their overall health [18]. Studies have highlighted an association between excessive screen time and disrupted sleep patterns, insufficient physical activity, and low academic performance [19–21].

Considering that 4.76 billion people across the globe are using social media, representing 59.4% of the global population [22], and that 90% of Spanish adolescents utilize social networks, alongside the rising prevalence of mental health issues in this demographic over the past years [23, 24], it becomes crucial to examine how these habits might affect psychosocial well-being in this population. By shedding light on the potential risks and consequences of excessive screen time and digital communication, this research seeks to inform public health interventions, educational programs, and policy initiatives aimed at promoting responsible digital citizenship and safeguarding the health and development of young people in the digital age. Therefore, this study aimed to verify the associations between the use of social networks, messaging applications, and the addiction to social networks with psychosocial health in Spanish adolescents.

## Methods

### Study design and sample

This is a secondary cross-sectional study with data from the Eating Healthy and Daily Life Activities (EHDLA) study. This investigation involved a representative group of adolescents aged 12 to 17 years residing in the *Valle de Ricote*, within the Region of Murcia (Spain). It was conducted across their three secondary schools during the 2021/2022 academic year. The comprehensive methodology of the EHDLA research is documented elsewhere [25]. The research project received ethical approval from two committees, the Bioethics Committee of the University of Murcia (ID 2218/2018) and the Ethics Committee of the Albacete University Hospital Complex and the Albacete Integrated Care Management (ID 2021–85). Also, the study adhered to the principles outlined in the Helsinki Declaration, prioritizing the safeguarding of participants’ human rights.

For the present study, 632 adolescents (50.8%) with complete information on all variables of interest were included. The following inclusion criteria were considered: fall within the age range of 12 to 17 years and reside in or attend school in *Valle de Ricote*. Exclusion criteria were as follows: being excused from physical education classes, as assessments and surveys were conducted during these sessions; having any medical condition limiting physical activity or necessitating special care; undergoing any pharmacological treatment; or absence of parental or legal guardian consent.

To take part in this study, parents or legal guardians of the adolescents were required to sign an informed consent form. Furthermore, both parents/legal guardians and their children received an informational document detailing the study’s objectives, assessment instruments, and questionnaires used. Moreover, adolescents were explicitly invited to indicate their willingness to participate in the study.

### Measurements

#### Social network use

The assessment of social network use (Facebook, Twitter, Instagram, Snapchat, and TikTok) involved evaluating each social network individually using a single-item scale (“Please, indicate the option that you consider most appropriate for yourself regarding the use of each social network of the following:”). Adolescents were asked to indicate their usage level for each social network from five response options: (a) “I never or rarely use them”; (b) “I am a low consumer”; (c) “I am a medium consumer”; (d) “I am a fairly high consumer”; or “I am a very high consumer” [26]. The responses were transformed into numerical variables ranging from 1 (“I never or rarely use them”) to 5 (“I am a very high consumer”). Subsequently, the scores for each social network were summed to generate a social network use score, ranging from 5 to 25, with higher scores indicating greater social network use [27]. Given the absence of specific cutoff points for social network use, the social network use score was stratified into tertiles: low social network use (5 to 12 points), moderate social network use (13 to 15 points), and high social network use (16 to 25 points).

The WhatsApp use (i.e., a messaging application) was also assessed using the same single-item scale. However, given that WhatsApp is a messaging application rather than a social network, it was excluded from the social network use score calculation.

For further analyses, the use of each social network and WhatsApp were categorized into: (a) low social network use (“I never or rarely use them” or “I am a low consumer”); (b) medium social network use (“I am a medium consumer”); or (c) high social network use (“I am a fairly high consumer” or “I am a very high consumer).

#### Addictive behaviors to social network use

The Short Social Networks Addiction Scale-6 Symptoms (SNAddS-6 S) [28] was employed to assess potential addiction to social networks. This instrument comprises six items capturing behaviors associated with tolerance (i.e., a desire for increased use), salience (i.e., social network use becoming a primary concern), mood modification (i.e., altering mood through social network usage), relapse (i.e., the risk of returning to addiction after controlling use), withdrawal (i.e., experiencing psychological and physical symptoms when unable to use), and conflict (i.e., social network usage interfering with social and daily activities). It features a unifactorial structure and has been previously validated among Spanish adolescents [28]. To facilitate further analysis, responses for all these behaviors were aggregated (“No” = 0; “Yes” = 1) to derive an overall score for social network addictive behaviors (ranging from 0 to 6 behaviors), with higher scores indicating greater susceptibility to social network addiction. Additionally, the overall addictive-related behaviors to social network use were stratified into tertiles: low addictive behaviors (0 to 1 behavior), moderate addictive behaviors (2 to 3 behaviors), or high addictive behaviors (4 to 6 behaviors).

#### Psychosocial health problems

The psychosocial health was assessed using the 25-item self-report version of the Strengths and Difficulties Questionnaire (SDQ) [29]. This tool is utilized for clinical assessment, screening of psychiatric disorders, and epidemiological research. The SDQ comprises five scales: (i) emotional symptoms, (ii) conduct problems, (iii) hyperactivity, (iv) peer problems, and (v) pro-social behavior (reverse scored). Participants respond to the 25 items using a 3-point scale: “certainly true”, “somewhat true”, and “not true”, with scores ranging from 0 to 2 points. To calculate the SDQ score, all the scales are added together except for the prosocial scale, so the score ranges from 0 to 40 points. Additionally, cutoff scores were employed to categorize individuals into three groups: (a) normal (0–15 points); (b) borderline (16–19 points); and (c) abnormal (20–40 points) [29]. For further analyses, these groups were collapsed into: no psychosocial health problem (“normal” or “borderline”) or psychosocial health problems (“abnormal”).

#### Covariates

The adolescents provided self-reported data on their age and sex. Furthermore, socioeconomic status was assessed using the Family Affluence Scale (FAS-III) [30], comprising six questions with responses graded from 0 to 13 points. The cumulative scores were computed to determine the FAS-III score, with higher values indicating greater socioeconomic status.

The body weight and height of the adolescents were measured, and afterward, body mass index (BMI) was calculated by dividing their weight in kilograms by their height in meters squared.

To collect data on physical activity and sedentary behavior among adolescents, the Spanish version of the Youth Activity Profile (YAP-S) was applied, which was adapted and validated for its implementation among Spanish youth [31]. Additionally, participants’ sleep patterns were assessed by eliciting their typical weekday and weekend bedtime and wake-up times separately. The mean daily sleep duration for each participant was calculated using the formula: [(average nocturnal sleep duration on weekdays × 5) + (average nocturnal sleep duration on weekends × 2)]/7.

**Table 1:** Descriptive data of the study participants (*N* = 632)

Variables		Social networks use (status)		
		Low	Medium	High
Age (years)	Median (IQR)	13.0 (3.0)	14.0 (2.0)	14.0 (2.0)
Sex	Boys (%)	129 (49.2)	81 (42.0)	62 (35.0)
	Girls (%)	133 (50.8)	112 (58.0)	115 (65.0)
FAS-III (score)	Median (IQR)	8.0 (3.0)	8.0 (3.0)	9.0 (3.0)
Overall sleep duration (minutes)	Median (IQR)	514.3 (64.3)	497.1 (64.3)	480.0 (68.6)
YAP-S physical activity (score)	Median (IQR)	2.7 (0.8)	2.5 (0.8)	2.5 (1.0)
YAP-S sedentary behavior (score)	Median (IQR)	2.4 (0.8)	2.6 (0.8)	2.6 (1.0)
BMI (kg/m^2^)	Median (IQR)	21.3 (5.9)	22.1 (5.7)	22.0 (5.4)
KIDMED (score)	Median (IQR)	7.0 (4.0)	7.0 (3.0)	6.0 (3.0)
Facebook use (score)	Median (IQR)	1.0 (0.0)	1.0 (0.0)	1.0 (0.0)
Twitter use (score)	Median (IQR)	1.0 (0.0)	1.0 (0.0)	2.0 (2.0)
Instagram use (score)	Median (IQR)	3.0 (1.0)	4.0 (1.0)	5.0 (1.0)
Snapchat use (score)	Median (IQR)	3.0 (2.0)	4.0 (1.0)	5.0 (1.0)
TikTok use (score)	Median (IQR)	1.0 (0.0)	1.0 (0.0)	1.0 (.0)
SN use (score)	Median (IQR)	10.5 (3.0)	14.0 (2.0)	17.0 (2.0)
WhatsApp use (score)	Median (IQR)	2.0 (2.0)	4.0 (2.0)	5.0 (1.0)
Addictive behaviors to SN use (number)	Median (IQR)	1.0 (2.0)	2.0 (3.0)	3.0 (2.0)
Tolerance	Yes (%)	111 (42.4)	145 (75.1)	144 (81.4)
Salience	Yes (%)	42 (16.0)	49 (25.4)	61 (63.3)
Mood modification	Yes (%)	100 (38.2)	88 (45.6)	102 (57.6)
Relapse	Yes (%)	52 (19.8)	87 (45.1)	81 (45.8)
Withdrawal	Yes (%)	30 (11.5)	56 (29.0)	65 (36.7)
Conflict	Yes (%)	36 (13.7)	50 (28.2)	145 (22.9)
SDQ (score)^†^	Median (IQR)	9.5 (7.0)	13.0 (9.0)	12.0 (9.0)
SDQ status	Normal (%)	223 (85.1)	132 (68.4)	115 (65.0)
	Borderline (%)	22 (8.4)	24 (12.4)	33 (18.6)
	Abnormal (%)	17 (6.5)	37 (19.2)	29 (16.4)

*BMI* body mass index, *FAS-III* family affluence scale-III, *IQR* interquartile range, *KIDMED* Mediterranean diet quality index for children and adolescents, *SDQ* strengths and difficulties questionnaire, *SN* social network, *YAP-S* Spanish Youth Active Profile

^†^Sum of all scales except prosocial scale

The adherence to the Mediterranean Diet among children and adolescents was evaluated using the Mediterranean Diet Quality Index (KIDMED) [32]. This index consists of 16 questions regarding the frequency of consumption of healthy foods (e.g., fruits, vegetables) and unhealthy foods (e.g., sweets, pastries), as well as behaviors such as skipping breakfast or eating at fast-food restaurants. The total score ranges from − 4 to 12, with higher scores indicating better adherence to the Mediterranean Diet.

The inclusion of these covariates as adjustments is justified, given their relationship with psychosocial health. The literature demonstrates that adolescents from families with higher socioeconomic status tend to show better indicators of mental health [33]. Also, studies have shown associations between healthy eating habits, adequate sleep patterns, and regular physical exercise with better mental health among young people [33–35]. On the other hand, prolonged sedentary behaviors and overweight are often associated with higher odds of psychosocial problems, such as anxiety and depression [36, 37].

### Statistical analysis

To evaluate the normal distribution of the variables, we utilized visual methods like density and quantile–quantile plots, along with conducting the Shapiro-Wilk test. Therefore, continuous variables are shown as median and interquartile range (IQR), and categorical variables are shown as counts and percentages. Generalized linear regression models with binomial distribution were conducted to calculate the odds ratio (ORs) and their 95% confidence interval (CIs) for the associations between social network use status and psychosocial health problems (Supplementary material 1). Moreover, we calculated the predictive probabilities of having psychosocial health problems based on social network status or addiction to social network status. The models were adjusted for age (in years), sex (boys or girls), socioeconomic status (i.e., FAS-III score), physical activity (i.e., YAP-S physical activity score), sedentary behavior (i.e., YAP-S sedentary behavior score), overall sleep duration (in minutes), BMI (kg/m^2^), and adherence to the Mediterranean diet (i.e., KIDMED score). These same analyses were conducted for each social network (i.e., Facebook, Twitter, Instagram, Snapchat, and TikTok), a messaging application (i.e., WhatsApp), and for each addictive behavior to social networks (i.e., tolerance, salience, mood modification, relapse, withdrawal, and conflict). We carried out all the statistical analyses using R statistical software (version 4.3.2) (R Core Team, Vienna, Austria) and RStudio (version 2023.09.1 + 494) (Posit, Boston, MA, USA). We considered a *p* value less than 0.05 to be the threshold for statistical significance.

**Fig. 1:**
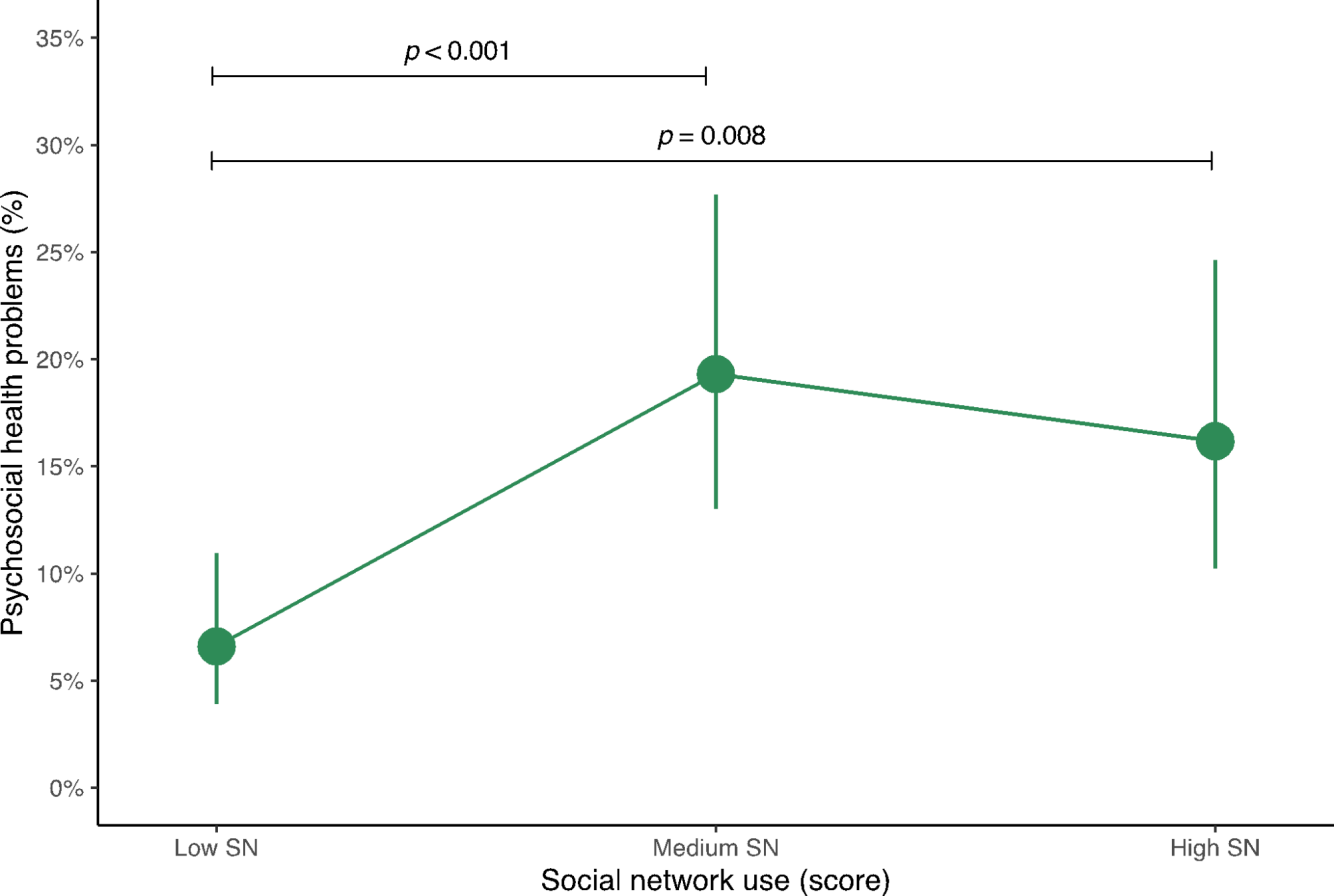
Predictive probabilities of having psychosocial health problems according to social network use status in adolescents. The data are expressed as predicted probabilities and 95% confidence intervals. Analyses were adjusted for age, sex, socioeconomic status, sleep duration, physical activity, sedentary behavior, body mass index, and adherence to the Mediterranean diet. CI, confidence interval; SN, social network. ^†^According to the Strengths and Difficulties Questionnaire (SDQ) [29]. ^‡^SDQ scores of 17 and above were considered as psychosocial health problems [29]

**Fig. 2:**
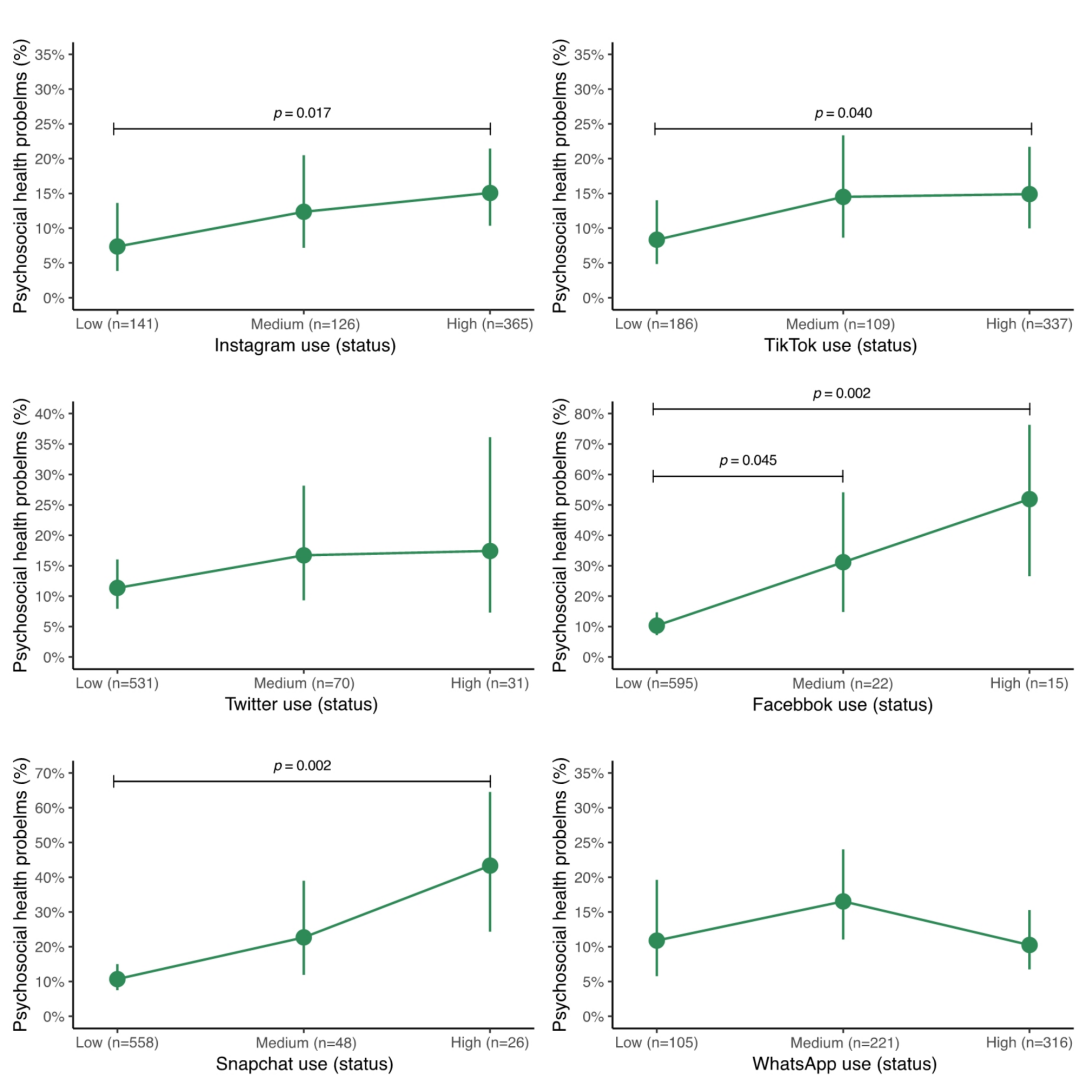
Predictive probabilities of having psychosocial health problems for each social network used or for WhatsApp use in adolescents. The data are expressed as predicted probabilities and 95% confidence intervals. Analyses were adjusted for age, sex, socioeconomic status, sleep duration, physical activity, sedentary behavior, body mass index, and adherence to the Mediterranean diet. CI, confidence interval. ^†^According to the Strengths and Difficulties Questionnaire (SDQ) [29]. ^‡^SDQ scores of 17 and above were considered as psychosocial health problems [29]. ^a^Significant difference from “low social network use” (*p* < 0.05)

**Table 2:** Predictive probabilities of having psychosocial health problems for each addictive behavior to social network use reported in adolescents

	Outcome^†^	
	Psychosocial health problems^‡^	
Predictors	% (95% CI)	*p*-value
Salience		
No	11.1 (7.2 to 16.9)	0.428
Yes	19.2 (9.1 to 19.2)	
Tolerance		
No	10.2 (7.0 to 14.6)	0.007
Yes	19.6 (12.9 to 28.6)	
Mood modification		
No	6.2 (3.8 to 10.0)	< 0.001
Yes	23.6 (16.6 to 32.4)	
Relapse		
No	9.9 (6.8 to 14.3)	0.004
Yes	19.0 (12.7 to 27.3)	
Withdrawal		
No	9.5 (6.5 to 13.7)	< 0.001
Yes	25.0 (16.7 to 35.6)	
Conflict		
No	9.0 (6.1 to 17.1)	< 0.001
Yes	25.2 (13.1 to 35.5)	

The data are expressed as predicted probabilities and 95% confidence intervals. Analyses were adjusted for age, sex, socioeconomic status, sleep duration, physical activity, sedentary behavior, body mass index, and adherence to the Mediterranean diet. CI, confidence interval

^†^According to the Strengths and Difficulties Questionnaire (SDQ) [29]

^‡^SDQ scores of 17 and above were considered as psychosocial health problems [29]

## Results

Table 1 presents the characteristics of the study participants according to social network use status. The highest proportion of individuals with a normal psychosocial health status (emotional symptoms, conduct problems, hyperactivity, peer problems, and pro-social behavior) was observed among participants with low social networking usage, while the lowest was among those with high social networking usage.

Figure 1 shows the predictive probabilities of having psychosocial health problems according to the social network use status in adolescents. The highest probability of presenting psychosocial health problems was identified in those who were classified as medium users (19.3%; 95% CI 13.0 to 27.7) and high users of social networks (16.2%; 95% CI 10.2 to 24.6). Conversely, the lowest probability of having psychosocial health problems were observed in those categorized as low users (6.6%; 95% CI 3.9 to 11.0). Moreover, significant differences were found between adolescents with low SN use and those with high SN use (*p* = 0.008), as well as with those with medium SN use (*p* < 0.001).

The predictive probabilities of having psychosocial health problems for each social network used or for WhatsApp use in adolescents are shown in Fig. 2 and Supplementary material 2. The probability of having psychosocial health problems was higher for those adolescents with high Instagram use 15.1% (95% CI 10.3 to 21.4), high TikTok use 14.9% (95% CI 10.0 to 21.7), high Snapchat use 43.3% (95% CI 24.3 to 64.5), compared to those with low use of these same social networks. Regarding Facebook, both medium and high users presented a higher probability of having psychosocial health problems compared to low users, 31.2% (95% CI 14.8 to 54.2) and 51.9% (95% CI 26.5 to 76.3), respectively. On the other hand, WhatsApp and Twitter were not significantly associated with psychosocial health problems.

Table 2 presents the predictive probabilities of psychosocial health problems associated with indicators of addictive behaviors to social network use among adolescents. Data indicate that adolescents reporting a desire to increase social network use (tolerance) have a 19.6% (95% CI 12.9 to 28.6) probability of experiencing such problems compared to the ones not reporting this desire. Similarly, those indicating mood alteration through social network usage (mood modification) show a 23.6% (95% CI 16.6 to 32.4) probability of encountering psychosocial health problems. Additionally, adolescents reporting a risk of relapse into addiction after attempting to control usage display a 19.0% (95% CI 12.7 to 27.3) probability of such problems. Those experiencing psychosocial and physical symptoms when unable to use social networks had a 25.0% (95% CI 16.7 to 35.6) probability of encountering psychosocial health issues. Lastly, adolescents reporting interference with social and daily activities due to social network usage present a 25.2% (95% CI 13.1 to 35.5) probability of experiencing psychosocial health problems.

**Fig. 3:**
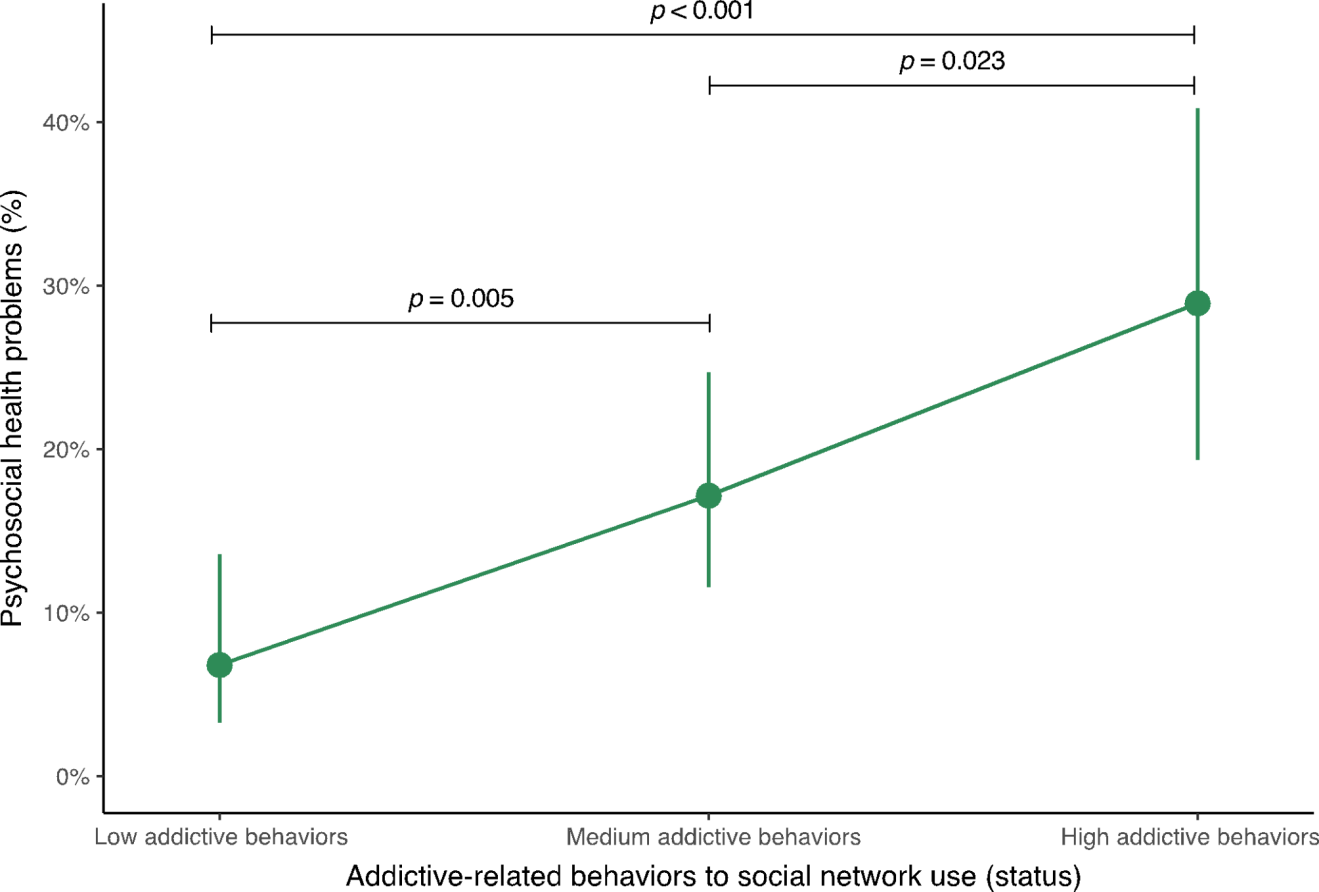
Predictive probabilities of having psychosocial health problems according to the addictive behaviors to social network use (status)

The predictive probabilities of having psychosocial health problems according to the addictive behaviors to social network use status in adolescents are found in Fig. 3 and Supplementary material 2. The highest probabilities of having psychosocial health problems were identified in those with high addictive behaviors to the SN use (28.9%; 95% CI 19.3 to 40.8%). Conversely, the lowest probabilities of having these same problems were observed in adolescents with low addictive behaviors (6.8%; 95% CI 3.3 to 13.6%). Furthermore, significant differences were found between adolescents with high addictive behaviors to SN use and those with low addictive behaviors to SN use (*p* < 0.001), as well as with those with medium addictive behaviors to SN use (*p* = 0.023). Likewise, significant differences were found between adolescents with medium addictive behaviors to SN use and those with low addictive behaviors to SN use (*p* = 0.005).

## Discussion

The key findings of this study revealed that medium users and high users of social media had higher probabilities of experiencing psychosocial health issues at 19.3 and 16.2% compared to lower users, respectively. Regarding the individual social network, Facebook emerged as the most detrimental, with 31.3% probability of psychosocial health problems among medium users and 51.9% among high users, followed by Snapchat users at 43.3%. High usage of Instagram and TikTok usage were also associated with approximately 15% probability of psychosocial health problems. Our data also revealed that adolescents exhibited various signs of social network addiction, such as tolerance, mood alteration, risk of relapse, withdrawal symptoms, and interference with daily activities, having probabilities ranging from 19.0 to 25.2% of experiencing psychosocial health problems. These associations were observed considering the role of several relevant covariates. Finally, we also noticed that the highest probabilities of having psychosocial health problems were identified in adolescents with high addictive behaviors when using social networks and the lowest in those with low addictive behaviors.

To the best of our knowledge, this is the first study to investigate these issues in the adolescent population from Spain, approaching separately the role of different social media platforms. Taken together, these findings underscore a concerning scenario regarding the use of social network and psychosocial health of Spanish adolescents. The results suggest that harm to psychosocial well-being is evident even with moderate use of social networks, indicating that it is not solely high usage that is associated with a risk to adolescents’ health. Previous literature has shown that excessive social network use (more than 3 h a day) is linked to psychosocial distress and internalizing problems among adolescents [8, 38]. However, our findings suggest that even moderate use of social networks is associated with worse psychosocial health, suggesting that the impact of social media on psychosocial well-being may be more pronounced than previously thought. On the other hand, our findings indicate that low usage of social networks is associated with a lower probability of experiencing psychosocial health issues, suggesting that abstaining from using social networks may prevent psychosocial health problems.

In this context, it is essential to consider the role of the different social networks, due to their individual characteristics. Each platform offers unique features and content, which could influence how adolescents engage with them and the potential impact on their psychosocial well-being [5]. We observed that Facebook and Snapchat were the social networks most strongly associated with psychosocial health problems. What is shown by existing literature is that, while it may be beneficial for some aspects like emotional support, self-expression, self-identity, and real-world relationships [39, 40], prolonged use of Facebook and Snapchat by adolescents is also associated with symptoms of anxiety and depression, loneliness, sleep problems, body image issues, and bullying [39, 41–44]. These adverse effects could be attributed to tendencies toward comparison and hopelessness, nighttime exposure to screens inhibiting the release of sleep hormones, instant propagation of negative comments, and the perception that by observing others’ “exciting” lives, they are missing out on the chance to live their own [45, 46]. On the other hand, the psychological necessities of adolescents may motivate the search for content that will bring relief to their symptoms [47].

Another aspect identified was the addictive behaviors to social network use among adolescents and their relationship with psychosocial health problems. Each indicator of social media addiction presents unique implications for adolescent well-being. Tolerance, manifested as a desire to increase social media use, signifies a growing dependence on digital platforms for social interaction and validation, potentially leading to escalated addictive behaviors over time [48]. Similarly, mood alteration through social media use reflects the emotional impact of online interactions, suggesting that the immersive nature of social media platforms could influence adolescents’ mood and overall psychological well-being [49]. Moreover, the risk of relapse into addiction after attempting to control use underscores the challenges teens face in regulating their online behavior, highlighting the dependence they develop on digital platforms for emotional support, social connection and continuous reward [50]. Additionally, interference with social and daily activities due to social media use disrupts teenagers’ offline lives, exacerbating feelings of isolation and detachment from real-world interactions, ultimately compromising their overall functioning and well-being [51]. These findings underscore the multifaceted nature of social media addiction and its profound impact on various dimensions of adolescent psychosocial health.

Several mechanisms could explain the link between social media use and psychosocial health problems. Factors such as idealization of a perfect life, constant comparison, cyberbullying, body image comparisons, and disrupted sleep patterns due to late-night usage all play a role [52, 53]. Also, evidence indicates that social media use can lead to dopamine release in the brain, similar to other addictive behaviors. Dopamine is a neurotransmitter associated with pleasure and reward. When we engage in activities that we find pleasurable, such as receiving likes or comments on social media posts, dopamine is released in our brains, reinforcing the behavior and encouraging us to continue engaging in it [54, 55]. Another aspect involved is that excessive social media use can lead to dysregulation of dopamine pathways, resulting in individuals feeling compelled to check their social media accounts frequently, even when it interferes with other aspects of their lives such as work, sleep, or social interactions [55]. It is also speculated that this relationship may be bidirectional, meaning that having symptoms led to higher social media usage [47].

Regarding addiction to social network behavior predicting psychosocial health problems, the literature presents similar results. Aspects such as attention deficit hyperactivity disorder, anxiety, and depression are associated with social media dependency, demonstrating in some cases that the greater the use of social media, the more severe the symptoms [56, 57]. Sümen and Evgin [58] pointed out that Turkish adolescents who were addicted to social media had a higher risk of developing various mental health issues, such as conduct problems, emotional issues, and issues with peers. Possible mechanisms for this relationship were also indicated, including the barrier that social networks create to establishing personal relationships with family and the environment; the use of social media to relieve undesirable mood states, symptoms of stress and anxiety, and even social media as a mechanism for modulating neuroendocrine responses and the sympathetic nervous system in the face of a stressor event [56–58].

There are some limitations to consider in this study. First, the use of social network and psychosocial health were based on self-reports by adolescents, which could potentially lead to super estimation or underestimation of data, depending on memory, and may be affected by social desirability. Also, the lack of predefined thresholds for social media usage levels may introduce variability in how participants interpret and report their usage. Additionally, as social media usage patterns evolve rapidly, our data, while collected relatively recently, may not capture the most current trends and newest platforms. Finally, the sample was not representative, which can make it difficult to extrapolate the results. On the other hand, some important strengths of this study must be considered. Our study examined multiple social media platforms and their individual impact, offers crucial insights for developing targeted interventions and prevention strategies. Furthermore, we included in the analyses a large number of covariates, such as age, sex, socioeconomic status, sleep duration, physical activity, sedentary behavior, BMI, and adherence to the Mediterranean diet, thereby enhancing the validity of the present results.

In conclusion, adolescents who use social networks more frequently and exhibit more addictive behaviors related to their use are more likely to experience psychosocial health problems compared to those who do not.Facebook emerged as the most detrimental, followed by Snapchat, Instagram, and TikTok. Our data also revealed that adolescents exhibit various signs of social network addiction and that the greater the addictive behaviors, the greater the psychosocial health problems. Taken together, the findings of the present study have the potential to contribute to a more nuanced understanding of the complex relationship between technology use and adolescent well-being, with implications for both research and practice in the fields of psychology, education, and public health.

### Supplementary Information

Supplementary material 1

Supplementary material 2

## Data Availability

The data used in this study are available upon request from the corresponding authors. However, given that the participants are minors, privacy and confidentiality must be respected.
