# Supplementary material for: Scrolling through adolescence: unveiling the relationship of the use of social networks and its addictive behavior with psychosocial health
Source: Child Adolesc Psychiatry Ment Health. 2024 Aug 31;18:107. doi: 10.1186/s13034-024-00805-0 (PMC11365153; doi:10.1186/s13034-024-00805-0)
Supplement: Supplementary file 1 — Supplementary material 1 [file 13034_2024_805_MOESM1_ESM.docx]

# Supplementary material 1

# Table S1. Association of social network use and covariates with psychosocial health problems in adolescents.

| Predictor | OR | SE | LLCI | ULCI | *p*-value |
| --- | --- | --- | --- | --- | --- |
| Low SN use | Reference |  |  |  |  |
| Medium SN use | 3.26 | 0.34 | 1.69 | 6.31 | <0.001 |
| High SN use | 2.63 | 0.36 | 1.30 | 5.32 | 0.007 |
| Boys | Reference |  |  |  |  |
| Girls | 0.92 | 0.27 | 0.54 | 1.56 | 0.746 |
| Age (per one year) | 0.88 | 0.09 | 0.73 | 1.05 | 0.147 |
| FAS-III score (per one point) | 0.92 | 0.06 | 0.81 | 1.03 | 0.152 |
| YAP-S physical activity (per one point) | 1.31 | 0.19 | 0.90 | 1.92 | 0.159 |
| YAP-S sedentary behaviors (per one point) | 1.07 | 0.22 | 0.70 | 1.64 | 0.752 |
| Overall sleep duration (per one hour) | 0.75 | 0.14 | 0.57 | 1.00 | 0.049 |
| BMI (per one kg/m^2^) | 1.02 | 0.03 | 0.96 | 1.07 | 0.543 |
| KIDMED score (per one point) | 0.93 | 0.05 | 0.83 | 1.03 | 0.149 |

BMI, body mass index; KIDMED, Mediterranean Diet Quality Index in children and adolescents; LLCI, lower limit confidence interval; OR, odds ratio; SE, standard error; SN, social network; ULCI, upper limit confidence interval; YAP-S, Spanish Youth Active Profile.

# Table S2. Association of addictive behaviors to social network use status and covariates with psychosocial health problems in adolescents.

| Predictor | OR | SE | LLCI | ULCI | *p*-value |
| --- | --- | --- | --- | --- | --- |
| Low addictive behaviors | Reference |  |  |  |  |
| Medium addictive behaviors | 3.34 | 0.49 | 1.28 | 8.71 | 0.014 |
| High addictive behaviors | 7.10 | 0.49 | 2.70 | 18.63 | <0.001 |
| Boys | Reference |  |  |  |  |
| Girls | 0.68 | 0.29 | 0.38 | 1.19 | 0.175 |
| Age (per one year) | 0.86 | 0.10 | 0.71 | 1.04 | 0.113 |
| FAS-III score (per one point) | 0.96 | 0.06 | 0.85 | 1.09 | 0.511 |
| YAP-S physical activity (per one point) | 1.23 | 0.20 | 0.83 | 1.82 | 0.295 |
| YAP-S sedentary behaviors (per one point) | 0.94 | 0.24 | 0.59 | 1.49 | 0.787 |
| Overall sleep duration (per one hour) | 0.79 | 0.15 | 0.58 | 1.06 | 0.115 |
| BMI (per one kg/m^2^) | 1.01 | 0.03 | 0.95 | 1.06 | 0.787 |
| KIDMED score (per one point) | 0.91 | 0.06 | 0.82 | 1.02 | 0.109 |

BMI, body mass index; KIDMED, Mediterranean Diet Quality Index in children and adolescents; LLCI, lower limit confidence interval; OR, odds ratio; SE, standard error; ULCI, upper limit confidence interval; YAP-S, Spanish Youth Active Profile.

# Table S3. Association of Instagram use and covariates with psychosocial health problems in adolescents.

| Predictor | OR | SE | LLCI | ULCI | *p*-value |
| --- | --- | --- | --- | --- | --- |
| Low Instagram use | Reference |  |  |  |  |
| Medium Instagram use | 1.58 | 0.46 | 0.63 | 3.91 | 0.327 |
| High Instagram use | 2.19 | 0.39 | 1.02 | 4.70 | 0.044 |
| Boys | Reference |  |  |  |  |
| Girls | 1.02 | 0.27 | 0.60 | 1.74 | 0.944 |
| Age (per one year) | 0.82 | 0.09 | 0.68 | 0.99 | 0.035 |
| FAS-III score (per one point) | 0.93 | 0.06 | 0.82 | 1.05 | 0.224 |
| YAP-S physical activity (per one point) | 1.21 | 0.19 | 0.83 | 1.77 | 0.325 |
| YAP-S sedentary behaviors (per one point) | 1.12 | 0.22 | 0.73 | 1.71 | 0.601 |
| Overall sleep duration (per one hour) | 0.71 | 0.14 | 0.53 | 0.94 | 0.016 |
| BMI (per one kg/m^2^) | 1.01 | 0.03 | 0.96 | 1.07 | 0.597 |
| KIDMED score (per one point) | 0.94 | 0.05 | 0.84 | 1.04 | 0.210 |

BMI, body mass index; KIDMED, Mediterranean Diet Quality Index in children and adolescents; LLCI, lower limit confidence interval; OR, odds ratio; SE, standard error; ULCI, upper limit confidence interval; YAP-S, Spanish Youth Active Profile.

# Table S4. Association of TikTok use and covariates with psychosocial health problems in adolescents.

| Predictor | OR | SE | LLCI | ULCI | *p*-value |
| --- | --- | --- | --- | --- | --- |
| Low TikTok use | Reference |  |  |  |  |
| Medium TikTok use | 1.80 | 0.42 | 0.79 | 4.14 | 0.163 |
| High TikTok use | 1.97 | 0.35 | 0.99 | 3.89 | 0.052 |
| Boys | Reference |  |  |  |  |
| Girls | 0.95 | 0.28 | 0.55 | 1.63 | 0.849 |
| Age (per one year) | 0.89 | 0.09 | 0.74 | 1.07 | 0.208 |
| FAS-III score (per one point) | 0.93 | 0.06 | 0.82 | 1.04 | 0.204 |
| YAP-S physical activity (per one point) | 1.29 | 0.19 | 0.88 | 1.88 | 0.189 |
| YAP-S sedentary behaviors (per one point) | 1.13 | 0.22 | 0.74 | 1.73 | 0.576 |
| Overall sleep duration (per one hour) | 0.72 | 0.14 | 0.54 | 0.95 | 0.020 |
| BMI (per one kg/m^2^) | 1.01 | 0.03 | 0.95 | 1.06 | 0.801 |
| KIDMED score (per one point) | 0.93 | 0.05 | 0.84 | 1.03 | 0.182 |

BMI, body mass index; KIDMED, Mediterranean Diet Quality Index in children and adolescents; LLCI, lower limit confidence interval; OR, odds ratio; SE, standard error; ULCI, upper limit confidence interval; YAP-S, Spanish Youth Active Profile.

# Table S5. Association of Twitter use and covariates with psychosocial health problems in adolescents.

| Predictor | OR | SE | LLCI | ULCI | *p*-value |
| --- | --- | --- | --- | --- | --- |
| Low Twitter use | Reference |  |  |  |  |
| Medium Twitter use | 1.49 | 0.38 | 0.71 | 3.12 | 0.286 |
| High Twitter use | 1.76 | 0.50 | 0.66 | 4.71 | 0.259 |
| Boys | Reference |  |  |  |  |
| Girls | 1.09 | 0.27 | 0.64 | 1.85 | 0.760 |
| Age (per one year) | 0.84 | 0.09 | 0.70 | 1.01 | 0.067 |
| FAS-III score (per one point) | 0.94 | 0.06 | 0.83 | 1.06 | 0.310 |
| YAP-S physical activity (per one point) | 1.23 | 0.20 | 0.84 | 1.81 | 0.286 |
| YAP-S sedentary behaviors (per one point) | 1.13 | 0.22 | 0.73 | 1.72 | 0.589 |
| Overall sleep duration (per one hour) | 0.71 | 0.14 | 0.54 | 0.93 | 0.014 |
| BMI (per one kg/m^2^) | 1.01 | 0.03 | 0.96 | 1.07 | 0.716 |
| KIDMED score (per one point) | 0.92 | 0.05 | 0.83 | 1.02 | 0.129 |

BMI, body mass index; KIDMED, Mediterranean Diet Quality Index in children and adolescents; LLCI, lower limit confidence interval; OR, odds ratio; SE, standard error; ULCI, upper limit confidence interval; YAP-S, Spanish Youth Active Profile.

# Table S6. Association of Facebook use and covariates with psychosocial health problems in adolescents.

| Predictor | OR | SE | LLCI | ULCI | *p*-value |
| --- | --- | --- | --- | --- | --- |
| Low Facebook use | Reference |  |  |  |  |
| Medium Facebook use | 3.97 | 0.52 | 1.44 | 10.94 | 0.008 |
| High Facebook use | 8.98 | 0.56 | 3.02 | 26.72 | <0.001 |
| Boys | Reference |  |  |  |  |
| Girls | 1.00 | 0.28 | 0.58 | 1.74 | 0.991 |
| Age (per one year) | 0.84 | 0.10 | 0.69 | 1.01 | 0.067 |
| FAS-III score (per one point) | 0.97 | 0.06 | 0.86 | 1.09 | 0.604 |
| YAP-S physical activity (per one point) | 1.22 | 0.20 | 0.82 | 1.81 | 0.320 |
| YAP-S sedentary behaviors (per one point) | 1.03 | 0.22 | 0.66 | 1.60 | 0.892 |
| Overall sleep duration (per one hour) | 0.66 | 0.15 | 0.50 | 0.88 | 0.004 |
| BMI (per one kg/m^2^) | 1.00 | 0.03 | 0.95 | 1.06 | 0.979 |
| KIDMED score (per one point) | 0.91 | 0.05 | 0.82 | 1.01 | 0.087 |

BMI, body mass index; KIDMED, Mediterranean Diet Quality Index in children and adolescents; LLCI, lower limit confidence interval; OR, odds ratio; SE, standard error; ULCI, upper limit confidence interval; YAP-S, Spanish Youth Active Profile.

# Table S7. Association of Snapchat use and covariates with psychosocial health problems in adolescents.

| Predictor | OR | SE | LLCI | ULCI | *p*-value |
| --- | --- | --- | --- | --- | --- |
| Low Snapchat use | Reference |  |  |  |  |
| Medium Snapchat use | 2.15 | 0.40 | 0.98 | 4.71 | 0.057 |
| High Snapchat use | 6.43 | 0.44 | 2.73 | 15.15 | <0.001 |
| Boys | Reference |  |  |  |  |
| Girls | 0.85 | 0.28 | 0.50 | 1.46 | 0.564 |
| Age (per one year) | 0.87 | 0.09 | 0.73 | 1.05 | 0.157 |
| FAS-III score (per one point) | 0.98 | 0.06 | 0.86 | 1.10 | 0.688 |
| YAP-S physical activity (per one point) | 1.23 | 0.20 | 0.84 | 1.82 | 0.289 |
| YAP-S sedentary behaviors (per one point) | 1.07 | 0.22 | 0.69 | 1.66 | 0.750 |
| Overall sleep duration (per one hour) | 0.70 | 0.14 | 0.53 | 0.93 | 0.014 |
| BMI (per one kg/m^2^) | 1.00 | 0.03 | 0.94 | 1.06 | 0.954 |
| KIDMED score (per one point) | 0.93 | 0.05 | 0.83 | 1.03 | 0.157 |

BMI, body mass index; KIDMED, Mediterranean Diet Quality Index in children and adolescents; LLCI, lower limit confidence interval; OR, odds ratio; SE, standard error; ULCI, upper limit confidence interval; YAP-S, Spanish Youth Active Profile.

# Table S8. Association of WhatsApp use and covariates with psychosocial health problems in adolescents.

| Predictor | OR | SE | LLCI | ULCI | *p*-value |
| --- | --- | --- | --- | --- | --- |
| Low WhatsApp use | Reference |  |  |  |  |
| Medium WhatsApp use | 1.53 | 0.38 | 0.73 | 3.24 | 0.261 |
| High WhatsApp use | 0.89 | 0.39 | 0.42 | 1.91 | 0.765 |
| Boys | Reference |  |  |  |  |
| Girls | 1.07 | 0.27 | 0.63 | 1.82 | 0.796 |
| Age (per one year) | 0.88 | 0.09 | 0.74 | 1.05 | 0.166 |
| FAS-III score (per one point) | 0.95 | 0.06 | 0.84 | 1.08 | 0.446 |
| YAP-S physical activity (per one point) | 1.22 | 0.19 | 0.83 | 1.77 | 0.308 |
| YAP-S sedentary behaviors (per one point) | 1.17 | 0.22 | 0.76 | 1.79 | 0.477 |
| Overall sleep duration (per one hour) | 0.70 | 0.14 | 0.53 | 0.92 | 0.010 |
| BMI (per one kg/m^2^) | 1.01 | 0.03 | 0.96 | 1.07 | 0.664 |
| KIDMED score (per one point) | 0.92 | 0.05 | 0.83 | 1.02 | 0.105 |

BMI, body mass index; KIDMED, Mediterranean Diet Quality Index in children and adolescents; LLCI, lower limit confidence interval; OR, odds ratio; SE, standard error; ULCI, upper limit confidence interval; YAP-S, Spanish Youth Active Profile.

# Table S9. Association of addictive behavior to social network use (tolerance) and covariates with psychosocial health problems in adolescents.

| Predictor | OR | SE | LLCI | ULCI | *p*-value |
| --- | --- | --- | --- | --- | --- |
| Tolerance (yes) | 1.21 | 0.29 | 0.69 | 2.12 | 0.508 |
| Boys | Reference |  |  |  |  |
| Girls | 1.05 | 0.27 | 0.62 | 1.79 | 0.859 |
| Age (per one year) | 0.86 | 0.09 | 0.72 | 1.03 | 0.106 |
| FAS-III score (per one point) | 0.94 | 0.06 | 0.83 | 1.06 | 0.289 |
| YAP-S physical activity (per one point) | 1.21 | 0.19 | 0.83 | 1.77 | 0.315 |
| YAP-S sedentary behaviors (per one point) | 1.15 | 0.22 | 0.75 | 1.76 | 0.520 |
| Overall sleep duration (per one hour) | 0.71 | 0.14 | 0.54 | 0.94 | 0.017 |
| BMI (per one kg/m^2^) | 1.01 | 0.03 | 0.96 | 1.06 | 0.738 |
| KIDMED score (per one point) | 0.92 | 0.05 | 0.83 | 1.02 | 0.122 |

BMI, body mass index; KIDMED, Mediterranean Diet Quality Index in children and adolescents; LLCI, lower limit confidence interval; OR, odds ratio; SE, standard error; ULCI, upper limit confidence interval; YAP-S, Spanish Youth Active Profile.

# Table S10. Association of addictive behavior to social network use (salience) and covariates with psychosocial health problems in adolescents.

| Predictor | OR | SE | LLCI | ULCI | *p*-value |
| --- | --- | --- | --- | --- | --- |
| Salience (yes) | 2.18 | 0.26 | 1.30 | 3.66 | 0.003 |
| Boys | Reference |  |  |  |  |
| Girls | 1.00 | 0.27 | 0.59 | 1.70 | 0.996 |
| Age (per one year) | 0.89 | 0.09 | 0.74 | 1.06 | 0.194 |
| FAS-III score (per one point) | 0.96 | 0.06 | 0.85 | 1.09 | 0.552 |
| YAP-S physical activity (per one point) | 1.23 | 0.19 | 0.84 | 1.80 | 0.282 |
| YAP-S sedentary behaviors (per one point) | 1.08 | 0.22 | 0.70 | 1.65 | 0.741 |
| Overall sleep duration (per one hour) | 0.72 | 0.14 | 0.55 | 0.95 | 0.022 |
| BMI (per one kg/m^2^) | 1.01 | 0.03 | 0.96 | 1.07 | 0.653 |
| KIDMED score (per one point) | 0.91 | 0.05 | 0.82 | 1.01 | 0.071 |

BMI, body mass index; KIDMED, Mediterranean Diet Quality Index in children and adolescents; LLCI, lower limit confidence interval; OR, odds ratio; SE, standard error; ULCI, upper limit confidence interval; YAP-S, Spanish Youth Active Profile.

# Table S11. Association of addictive behavior to social network use (mood modification) and covariates with psychosocial health problems in adolescents.

| Predictor | OR | SE | LLCI | ULCI | *p*-value |
| --- | --- | --- | --- | --- | --- |
| Mood modification (yes) | 5.17 | 0.32 | 2.77 | 9.62 | <0.001 |
| Boys | Reference |  |  |  |  |
| Girls | 0.92 | 0.29 | 0.52 | 1.62 | 0.772 |
| Age (per one year) | 0.82 | 0.10 | 0.68 | 0.99 | 0.041 |
| FAS-III score (per one point) | 0.93 | 0.06 | 0.82 | 1.06 | 0.276 |
| YAP-S physical activity (per one point) | 1.21 | 0.20 | 0.81 | 1.79 | 0.353 |
| YAP-S sedentary behaviors (per one point) | 1.20 | 0.23 | 0.77 | 1.88 | 0.416 |
| Overall sleep duration (per one hour) | 0.82 | 0.15 | 0.61 | 1.10 | 0.182 |
| BMI (per one kg/m^2^) | 1.01 | 0.03 | 0.96 | 1.07 | 0.652 |
| KIDMED score (per one point) | 0.92 | 0.05 | 0.83 | 1.03 | 0.138 |

BMI, body mass index; KIDMED, Mediterranean Diet Quality Index in children and adolescents; LLCI, lower limit confidence interval; OR, odds ratio; SE, standard error; ULCI, upper limit confidence interval; YAP-S, Spanish Youth Active Profile.

# Table S12. Association of addictive behavior to social network use (relapse) and covariates with psychosocial health problems in adolescents.

| Predictor | OR | SE | LLCI | ULCI | *p*-value |
| --- | --- | --- | --- | --- | --- |
| Relapse (yes) | 2.32 | 0.26 | 1.39 | 3.86 | 0.001 |
| Boys | Reference |  |  |  |  |
| Girls | 0.85 | 0.28 | 0.49 | 1.46 | 0.550 |
| Age (per one year) | 0.86 | 0.09 | 0.72 | 1.03 | 0.110 |
| FAS-III score (per one point) | 0.94 | 0.06 | 0.83 | 1.06 | 0.289 |
| YAP-S physical activity (per one point) | 1.21 | 0.20 | 0.82 | 1.77 | 0.338 |
| YAP-S sedentary behaviors (per one point) | 1.13 | 0.22 | 0.74 | 1.74 | 0.571 |
| Overall sleep duration (per one hour) | 0.70 | 0.14 | 0.53 | 0.93 | 0.014 |
| BMI (per one kg/m^2^) | 1.01 | 0.03 | 0.95 | 1.06 | 0.796 |
| KIDMED score (per one point) | 0.92 | 0.05 | 0.83 | 1.02 | 0.105 |

BMI, body mass index; KIDMED, Mediterranean Diet Quality Index in children and adolescents; LLCI, lower limit confidence interval; OR, odds ratio; SE, standard error; ULCI, upper limit confidence interval; YAP-S, Spanish Youth Active Profile.

# Table S13. Association of addictive behavior to social network use (withdrawal) and covariates with psychosocial health problems in adolescents.

| Predictor | OR | SE | LLCI | ULCI | *p*-value |
| --- | --- | --- | --- | --- | --- |
| Withdrawal (yes) | 3.48 | 0.27 | 2.07 | 5.85 | <0.001 |
| Boys | Reference |  |  |  |  |
| Girls | 0.81 | 0.28 | 0.47 | 1.41 | 0.466 |
| Age (per one year) | 0.85 | 0.09 | 0.71 | 1.02 | 0.085 |
| FAS-III score (per one point) | 0.97 | 0.06 | 0.86 | 1.10 | 0.606 |
| YAP-S physical activity (per one point) | 1.22 | 0.20 | 0.83 | 1.80 | 0.306 |
| YAP-S sedentary behaviors (per one point) | 0.99 | 0.23 | 0.63 | 1.55 | 0.971 |
| Overall sleep duration (per one hour) | 0.69 | 0.15 | 0.52 | 0.92 | 0.011 |
| BMI (per one kg/m^2^) | 1.02 | 0.03 | 0.97 | 1.08 | 0.410 |
| KIDMED score (per one point) | 0.95 | 0.06 | 0.85 | 1.06 | 0.367 |

BMI, body mass index; KIDMED, Mediterranean Diet Quality Index in children and adolescents; LLCI, lower limit confidence interval; OR, odds ratio; SE, standard error; ULCI, upper limit confidence interval; YAP-S, Spanish Youth Active Profile.

# Table S14. Association of addictive behavior to social network use (conflict) and covariates with psychosocial health problems in adolescents.

| Predictor | OR | SE | LLCI | ULCI | *p*-value |
| --- | --- | --- | --- | --- | --- |
| Conflict (yes) | 3.57 | 0.26 | 2.14 | 5.97 | <0.001 |
| Boys | Reference |  |  |  |  |
| Girls | 0.88 | 0.28 | 0.51 | 1.52 | 0.651 |
| Age (per one year) | 0.87 | 0.09 | 0.72 | 1.04 | 0.132 |
| FAS-III score (per one point) | 0.98 | 0.06 | 0.87 | 1.11 | 0.753 |
| YAP-S physical activity (per one point) | 1.28 | 0.20 | 0.87 | 1.89 | 0.201 |
| YAP-S sedentary behaviors (per one point) | 1.03 | 0.23 | 0.66 | 1.61 | 0.899 |
| Overall sleep duration (per one hour) | 0.70 | 0.15 | 0.52 | 0.93 | 0.013 |
| BMI (per one kg/m^2^) | 1.02 | 0.03 | 0.97 | 1.08 | 0.445 |
| KIDMED score (per one point) | 0.94 | 0.05 | 0.85 | 1.05 | 0.286 |

BMI, body mass index; KIDMED, Mediterranean Diet Quality Index in children and adolescents; LLCI, lower limit confidence interval; OR, odds ratio; SE, standard error; ULCI, upper limit confidence interval; YAP-S, Spanish Youth Active Profile.
