# Supplementary material for: Scrolling through adolescence: unveiling the relationship of the use of social networks and its addictive behavior with psychosocial health
Source: Child Adolesc Psychiatry Ment Health. 2024 Aug 31;18:107. doi: 10.1186/s13034-024-00805-0 (PMC11365153; doi:10.1186/s13034-024-00805-0)
Supplement: Supplementary file 2 — Supplementary material 2 [file 13034_2024_805_MOESM2_ESM.docx]

## Supplementary material 2

## Table S1. Predictive probabilities of having psychosocial health problems according to social network use or addictive behaviors to social network use status in adolescents.

|  | **Outcome** ^†^ |
| --- | --- |
|  | **Psychosocial health problems ^‡^** |
| **Predictor** | **Low use** |
|  | *% (95% CI)* |
| **Social network use (status)** |  |
| **Low** | 6.6 (3.9 to 11.0) |
| **Medium** | 19.3 (13.0 to 27.7) ^a^ |
| **High** | 16.2 (10.2 to 24.6) ^a^ |
| **Addictive behaviors to social network use (status)** |  |
| **Low** | 6.8 (3.3 to 13.6) |
| **Medium** | 17.2 (11.5 to 24.7) ^a^ |
| **High** | 28.9 (19.3 to 40.8) ^a,b^ |

The data are expressed as predicted probabilities and 95% confidence intervals. Analyses were adjusted for age, sex, socioeconomic status, sleep duration, physical activity, sedentary behavior, body mass index, and adherence to the Mediterranean diet. CI, confidence interval. ^†^ According to the Strengths and Difficulties Questionnaire (SDQ) [29]. ^‡^ SDQ scores of 17 and above were considered as psychosocial health problems [29]. ^a^ Significant differences from “low status” (*p* < 0.05). ^b^ Significant differences from “moderate status” (*p* < 0.05).

## Table S2. Predictive probabilities of having psychosocial health problems for each social network used or for WhatsApp use in adolescents.

|  | **Outcome** ^†^ |
| --- | --- |
|  | **Psychosocial health problems** ^‡^ |
| **Predictor** | *% (95% CI)* |
| **Instagram use (status)** |  |
| **Low** | 7.4 (3.9 to 13.6) |
| **Medium** | 12.4 (7.2 to 20.5) |
| **High** | 15.1 (10.3 to 21.4) ^a^ |
| **TikTok use (status)** |  |
| **Low** | 8.3 (4.8 to 14.0) |
| **Medium** | 14.5 (8.6 to 23.3) |
| **High** | 14.9 (10.0 to 21.7) ^a^ |
| **Twitter use (status)** |  |
| **Low** | 11.4 (7.9 to 16.0) |
| **Medium** | 16.7 (9.3 to 28.2) |
| **High** | 17.4 (7.3 to 36.1) |
| **Facebook use (status)** |  |
| **Low** | 10.3 (7.2 to 14.7) |
| **Medium** | 31.2 (14.8 to 54.2) ^a^ |
| **High** | 51.9 (26.5 to 76.3) ^a^ |
| **Snapchat use (status)** |  |
| **Low** | 10.7 (7.5 to 15.0) |
| **Medium** | 22.7 (11.9 to 39.0) |
| **High** | 43.3 (24.3 to 64.5) ^a^ |
| **WhatsApp use (status)** |  |
| **Low** | 10.9 (5.8 to 19.6) |
| **Medium** | 16.5 (11.0 to 24.0) |
| **High** | 10.3 (6.7 to 15.3) |

The data are expressed as predicted probabilities and 95% confidence intervals. Analyses were adjusted for age, sex, socioeconomic status, sleep duration, physical activity, sedentary behavior, body mass index, and adherence to the Mediterranean diet. CI, confidence interval. ^†^ According to the Strengths and Difficulties Questionnaire (SDQ)[29] . ^‡^ SDQ scores of 17 and above were considered as psychosocial health problems [29]. ^a^ Significant difference from “low use” (*p* < 0.05).
